# How to understand it: management of the painful shoulder following neurological injury

**DOI:** 10.1136/pn-2022-003576

**Published:** 2023-03-07

**Authors:** C Lakra, R Higgins, B Beare, R Farrell, S Ajina, S Burns, M Lee, O Swayne

**Affiliations:** 1 Department of Therapy and Rehabilitation, National Hospital for Neurology & Neurosurgery, UK; 2 Department Neuro-inflammation, Queen Square Institute of Neurology, University College London, UK; 3 Department of Orthopaedics, University College London, UK; 4 Department of Clinical and Movement Neurosciences, Queen Square Institute of Neurology, University College London, UK

## Abstract

Shoulder pain is a common symptom following neurological injury, particularly in the presence of hemiparesis. It can be disabling, lead to poor functional outcomes, and increase care costs. Its aetiology is multifactorial, and several pathologies typically contribute to a clinical presentation. Astute diagnostic skills and a multi-disciplinary approach are required to recognise accurately those which are clinically relevant, and to implement appropriate stepwise management.

In the absence of large clinical trial data, we aim to provide a comprehensive, practical and pragmatic overview of shoulder pain in patients with neurological conditions. We use available evidence as the basis of producing a management guideline, and take into account interdisciplinary opinions of Neurology, Rehabilitation Medicine, Orthopaedics and Physiotherapy.

## Introduction

**Figure 1:**
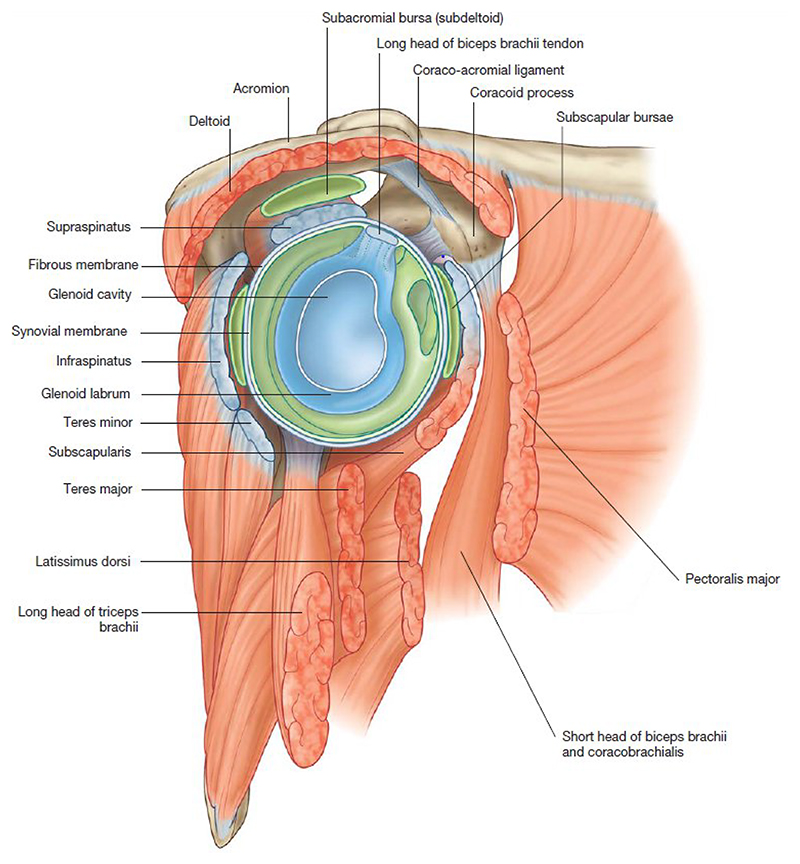
Anatomy of the shoulder^1^

Shoulder pain is a common and potentially disabling symptom in people following neurological injury. It refers to pain specifically experienced in the shoulder complex, which consists of several joints (glenohumeral (GHJ), acromioclavicular (ACJ), and sternoclavicular (SCJ)), alongside surrounding muscles, ligaments, cartilage, and bursae (Figure 1^1^). The physiological reasons for why pain develops are multifactorial and often involve a complex interaction between pathophysiological, psychological, and social factors.

The majority of patients with shoulder pain following stroke experience moderate to severe pain which can persist for many months.^2^ Shoulder pain poses a number of challenges to patients and their healthcare providers: it disturbs sleep, reduces participation in activities and rehabilitation, adversely affects quality of life and is associated with increased length of hospital stay.^3,4,5^ It inhibits movement^3^ and can subsequently lead to contracture formation and skin breakdown. Early proactive anticipation of risk and early management of shoulder pain is fundamental in preventing long-term complications and reducing care costs.

Published literature on this symptom has largely focused on the stroke population (referred to as ‘hemiplegic shoulder pain’ (HSP) or ‘post stroke shoulder pain’ (PSSP)). In the stroke population, prevalence estimates vary considerably; however, a recent systematic review found the prevalence at 12 months to be 39%.^6^ There is a paucity of large-scale published data related to other neurological conditions. Prevalence rates of 11% have been reported in people with Parkinson’s disease,^7^ 35% in multiple sclerosis,^8^ and 62% in traumatic brain injury in an inpatient setting.^9^ We suspect a degree of under-reporting occurs because of patient-related factors (cognitive and/or communication difficulties in expressing pain^10,11^), team-related factors (lack of awareness of the condition, variation in assessment methods), and/or system-related factors (lack of consistency in definition and diagnosis).

People with neurological conditions present with a number of possible impairments affecting the upper limb including reduced power, altered sensation and spasticity, all of which may predispose to the development of shoulder pain. Assessment and investigation will often reveal more than one diagnosis or pathology, not all of which may be clinically relevant. Moreover, patients with cognitive and/or communication deficits may be unable to participate fully in the assessment process, meaning conventional outcome measures of pain and function are often not appropriate for use in this group. Astute diagnostic skills and a multi-disciplinary approach are therefore required to recognise accurately the relevant pathologies and to optimise management.

The optimal methods to diagnose and manage shoulder pain in neurological populations remain contested in the literature, likely due to the heterogeneity of the condition and small sample sizes available for study. It is therefore unsurprising that even teams within the same organisation may have different approaches to care. We therefore aim to provide a comprehensive, practical and pragmatic overview of shoulder pain in patients with neurological conditions. Current available evidence is used as the basis of producing a management guideline, with a multi-specialty authorship including Neurology, Rehabilitation Medicine, Orthopaedics and Physiotherapy. In areas where research in this specific patient population is lacking, published findings of the non-neurological population have been used to guide recommendations where appropriate.

In our practice, shoulder pain in this patient group tends to present as one or more of four clinical presentations: hypotonia with subluxation, spasticity, sub-acromial pain syndrome (SAPS) and frozen shoulder. This paper will address the pathophysiology, diagnosis, and management of each, as well as providing guidance on protective handling, positioning, and pain management.

## Pathophysiology

The pathophysiology of shoulder pain can be considered in relation to the four common clinical presentations.

### Hypotonia and subluxation

Hypotonia and/or weakness of the rotator cuff muscles of the shoulder can lead to inferior subluxation of the humeral head within the joint. Subluxation in isolation may not in itself cause pain. However, a lack of support to the shoulder when handling or positioning the arm can lead to traction-related injuries including muscle tears, peripheral nerve damage, and overstretching of the peri-articular ligaments and joint capsule.^12,13^ Patients with severe hemiplegia and/or altered sensation may be at higher risk.^13^

### Spasticity

Spasticity is defined as intermittent or sustained involuntary muscle activity due to disrupted sensorimotor control as a result of an upper motor neurone lesion.^14^ Spasticity of the shoulder muscles typically affects the pectoralis major, subscapularis, latissimus dorsi and teres major.^3^ This pattern of muscle involvement results in an internally rotated and adducted resting position of the humerus and anterior subluxation of the humeral head. If left untreated, shortening of the affected muscles occurs, with subsequent progressive loss of shoulder joint range and associated pain.^15^ The abnormal positioning of the arm predisposes to the development of SAPS and therefore pain. Pain of any cause may drive an increase in spasticity, creating a negative feedback loop.

### Sub-acromial pain syndrome (SAPS)

SAPS is an umbrella term encompassing all non-traumatic, usually unilateral, shoulder problems that cause pain localised around the acromion.^16^ Conditions such as rotator cuff tendinopathy and tears, bursitis and biceps tendinopathy or tendonitis are commonly included under this term.^16^ Malalignment of the shoulder joint, such as in the presence of spasticity or an imbalance in strength of the rotator cuff muscles can lead to compression and/or irritation of soft tissues between the humeral head and coracoacromial arch.^12^ With the median age for stroke in the UK being 77 years,^17^ it is possible that tendons may have already undergone age-related collagen changes known as ‘tendinosis’ which makes these structures more susceptible to injury.^18^

### Frozen shoulder

Following neurological injury, local injury to the soft tissues of the shoulder can result in a pro-inflammatory environment. A complex cascade of events is triggered, including cytokine-mediated synovial inflammation and fibroblastic proliferation.^19^ Synovial hyperplasia, sub-synovial hypervascularity, and neurogenesis can occur.^20^ An increase in collagen deposition leads to thickening and contracture of the capsule and reduction of the volume of fluid within the capsule. This results in restricted range of motion, particularly that of external rotation. ^19,21,22^

Frozen shoulder is often associated with a ‘pain predominant’ early phase where this active inflammatory process results in pain during movement, at rest and at night. This is followed by a ‘stiffness predominant’ phase where restriction is present due to joint fibrosis but pain and possibly the inflammatory environment have reduced.^22^

### Other factors influencing shoulder pain

An important consideration in the presence of an underlying neurological condition is whether pain modulating centres are involved. The incidence of central post stroke pain (CPSP) has been found to be 18% in people with thalamic stroke^23^and 25% following medullary stroke.^24^ CPSP can result in misinterpretation of sensory inputs where patients may experience hypersensitivity, allodynia and burning pain.^25^ Patients are also at risk of developing Shoulder-Hand Syndrome (SHS), a variant of Complex Regional Pain Syndrome (CRPS).^26^

Higher centres such as the amygdala, anterior cingulate cortex and anterior insula feed directly into pain modulatory circuits and therefore anxiety, mood and past experiences may all influence pain perception. Lastly, referred pain from other sources such as the cervical spine or visceral sources ^27^ may contribute to pain in the shoulder and should be considered as part of the assessment.

## Risk factors

Risk factors for developing shoulder pain in the stroke population include: a high National Institutes of Health Stroke Scale item 5 score (corresponding to poorer motor control), the presence of upper limb spasticity, sensory impairment, restricted PROM of the shoulder, type 2 diabetes mellitus and a previous history of shoulder pain.^28,29^ Patients with one or more of these factors should be monitored as they are at risk of developing a painful shoulder.

## History and examination

A thorough history and comprehensive examination are essential in identifying the cause(s) of pain (Box 1).

## Investigations

The following imaging modalities may be used in conjunction with clinical assessment to help guide diagnosis:

⍰ Shoulder x-ray (true AP, scapular Y and axillary views): aids in the diagnosis of skeletal pathology such as; dislocation, osteoarthritis, fracture, osseous abnormalities, and heterotopic ossification.^30^ If shoulder subluxation is present, it can be characterised and measured.
⍰ Shoulder ultrasound: aids in the diagnosis of conditions such as bursitis, tendonitis, tendinosis calcarea, tears or partial tears. Ultrasound-guided steroid injection to the affected area(s) or to the GHJ may be arranged simultaneously.
⍰ Shoulder CT: to further assess fracture, arthropathy or osseous abnormalities such as dysplasia.
⍰ Shoulder MRI: this should be considered if intra-articular pathology or a partial/full rotator cuff tear is suspected.

## Diagnosis

The clinical characteristics and key assessment findings of the four clinical presentations are outlined in Box 2.

## Management

The physical management of all presentations involves appropriate manual handling and a plan for the 24-hour positioning of the affected limb (Box 3). In all cases, management should ideally be led by a multi-disciplinary team and be tailored to the individual’s functional status, quality of life, goals, and expected progress.^3^ Management should follow a stepwise approach and the effectiveness of each intervention should be reviewed at regular intervals. Specific physiotherapy and medical management recommendations of the four clinical presentations are outlined in Box 4. We recommend that first line treatments are continued alongside treatment escalations.

Steroid injections may be considered primarily for the management of acute exacerbations of pain not responding to first line treatments and simple analgesia, and/or in the context of meeting particular functional goals in the rehabilitation setting. They should not be considered routinely, nor do we advocate regularly repeated injections due to the potential risks of muscle atrophy and/or damage (for example tear or rupture). There are large variations between practitioners regarding how often and how many injections should be offered. A rough guide would be to repeat up to twice in total in the same location in cases of chronic intractable shoulder pain. Where steroids are mentioned in this paper, treatment is expected to show an effect 1-week post injection and to last 6-8 weeks.

When considering referral to the orthopaedic team, it is important to consider the background neurological injury and degree of functional recovery, hence the likelihood that surgery would offer benefit.

## Pain management

Due to the multi-factorial nature of shoulder pain following neurological injury, patients may experience one or more types of pain (broadly speaking nociceptive or neuropathic). Pain may be experienced in the shoulder itself or may be referred lower down the arm. Symptoms may occur at rest, on movement, and/or at night. There are several tools which may be used to quantify pain which take into account physical, cognitive, and/or communication impairments.^50,51^ These can be used to assess the effectiveness of treatment(s) over time. To assess for change following any intervention, we recommend that the same practitioners complete the tool(s) used for one patient.

The general management of pain in the shoulder is outlined in Box 5, which we recommend is used in conjunction with the above specific treatments of the common clinical presentations. Involvement of specialist pain management services should be considered. The goals and length of medications, as well as the risks and side effects, must be discussed with the patient and/or their carers before commencing. The responsible clinician should consider contra-indications of treatments in individual patients. Treatments should be started at low doses and up-titrated slowly, assessing for clinical response and side effects at regular intervals.

## Case vignette

A 60-year-old gentleman was admitted following the development of right sided hemiparesis, sensory impairment, global aphasia and collapse. He had a background of poorly controlled type 2 diabetes and bilateral lower limb amputations secondary to peripheral vascular disease. A CT head revealed the presence of a left thalamic ischaemic infarct. He was treated with dual antiplatelet therapy and once medically stable was transferred to a level 1 neuro-rehabilitation unit.

On the day of admission to the unit the patient was reviewed by the multi-disciplinary team, including a rehabilitation doctor, occupational therapist and physiotherapist. He reported ‘deep’ pain in his right shoulder on movement and at night. His local hospital had already commenced regular paracetamol and pregabalin 100mg twice per day. On examination he had clinical features of frozen shoulder with pain associated with reduced active and passive shoulder range of motion in all planes, particularly that of external rotation. Despite these findings he was able to use his right-hand for daily tasks including donning his prosthetic legs and self-propelling in his wheelchair. The team provided him with an individualised exercise programme which included shoulder stretches up to a pain-tolerable range of motion and strengthening exercises. He was encouraged to continue to use his right arm in daily tasks.

Shortly following admission, the patient was diagnosed with a non-ST elevation myocardial infarction and was transferred to an acute ward for one month. During this time, he had prolonged periods of bedrest and active rehabilitation was put on hold. On return to the neuro-rehabilitation unit, his shoulder pain had significantly worsened due to lack of use and immobility. He was now experiencing pain at rest, during movement and at night, causing him to wake several times. He was unable to use his arm functionally, nor engage in his exercise programme.

On examination, he had developed features of SHS with hand swelling, discolouration, cool skin, allodynia and hypersensitivity. He explained the pain radiated down his arm. The shoulder active and passive ranges of movement had also worsened (Table 1). The Scale of Pain Intensity (SPIN)^51^ was used to assess the severity of pain in the presence of aphasia (Table 2). An x-ray ruled out fracture, osteoarthritis and heterotopic ossification (which may have otherwise accounted for the stiffness).

The goals of treatment were to improve pain, particularly in relation to his upper limb therapy sessions, transfers, and daily activities. He was started on modified release morphine sulphate 20 mg twice a day and an additional short acting opiate as required and pregabalin was up-titrated. An ultrasound guided intra-articular GHJ steroid injection was performed using 5mL 2% lidocaine, 10mL 0.25% bupivacaine and 40 mg methylprednisolone.

The patient’s pain levels and PROM subsequently showed improvements (Tables 1 and 2). His symptoms of SHS also improved, however he was still struggling to tolerate therapy sessions and incorporate his arm into functional tasks. Following further multi-disciplinary review, a SSNB was undertaken which led to further improvements in his PROM and pain levels (Tables 1 and 2). Morphine was down-titrated prior to discharge, with a plan to review and aim to wean as an outpatient.

## Conclusions

Shoulder pain following neurological injury requires training and skill to recognise, diagnose and manage. Patients in this group may struggle to recognise and communicate their symptoms, given the coexistence of other physical, cognitive, and communicative impairments. Moreover, investigations may reveal additional pathologies, not all of which will be clinically relevant. Diagnosis therefore requires a toolkit of assessment methodologies ideally undertaken by a multidisciplinary team. Shoulder pain tends to present as one or more of four clinical presentations: hypotonia with subluxation, spasticity, sub-acromial pain syndrome (SAPS) and frozen shoulder. Early recognition and multi-disciplinary management could prevent long-term complications and reduce care costs.

## Figures and Tables

**Table 1:** Passive range of glenohumeral joint motion

Passive range of glenohumeral joint motion (degrees)	Return to Neurorehabilitation unit following medical instability	Post intra-articular steroid injection	Post SSNB
Flexion	55	80↑	130↑
Abduction	55	80↑	105↑
External rotation	10	20↑	30↑
Internal rotation	80	80↔	80↔

**Table 2:** Scale of Pain INtensity (SPIN) scores

Scale of Pain INtensity (SPIN) VAS scores 0-5 (0 is ‘no pain’, 5 is ‘pain as bad as it could be’)	Return to Neurorehabilitation following medical instability	Post intra-articular steroid injection	Post SSNB
Pain at rest	2	0↓	0↔
Pain on movement	5	4↓	2↓
Pain at night	2	0↓	0↔

## Box 1 Key points of a focussed history and examination of a painful shoulder

**History**

**Main complaint**

▪ Conduct a detailed pain history, including onset, severity, character, and whether pain occurs at rest, sleep, or movement
▪ The extent of function of the affected arm
▪ History of trauma if applicable
▪ Hand dominance

**Prior investigations related to the shoulder**

▪ May include x-ray, ultrasound, CT or MRI

**Prior management related to the shoulder**

▪ Review previous therapy and positioning techniques including orthotics and electrical stimulation
▪ Review previous interventions such as steroid injection or hydrodilatation
▪ Review pain medication

**Examination**

*Examine both shoulders for comparison*

**Inspection and palpation**

▪ Fully expose the neck, shoulders and upper limbs
▪ Inspect the resting alignment of the arms, shoulder joints, and scapula. Note asymmetry, wasting of muscles, and subluxation
▪ Measure extent and direction of subluxation
▪ Palpate and assess for pain in the SCJ, ACJ and GHJ
▪ Assess for allodynia and for features of shoulder-hand syndrome

**Tone**

▪ Note the presence/absence of increased tone. If spasticity present, identify which muscles are involved

**Range of movement**

▪ Active range of movement – this is the movement achieved by the patient lifting their own arm independently (if they are able). Assess for pain and restriction of range in shoulder flexion, abduction, internal and external rotation.
▪ Observe the quality of movement including co-ordination between the humerus and scapula (scapulohumeral rhythm^)12^
▪ Passive range of movement – this is the movement achieved by the assessor lifting the patient’s arm. Assess for pain and restriction of range in shoulder flexion, abduction, internal and external rotation. If shoulder subluxation is present, the assessment of range should be conducted with the assessor first re-aligning the humeral head in the GHJ.

**Power**

**Sensation**

**Neglect**

**Reflexes**

**Cervical spine examination**

**Other**

▪ Depending on your findings this may include shoulder special tests. But note: shoulder special tests have not been validated in the neurological population and a number of these tests require intact power to perform. They should be conducted with caution in patients presenting with subluxation and/or sensory impairment, as further injury or damage to the shoulder may occur with incorrect handling.

## Box 2 Clinical characteristics and key assessment findings of the four typical clinical presentations

**Hypotonia and subluxation**

**History**: pain may be felt at rest, particularly if the limb is unsupported, due to traction-like injury to soft tissues and/or peripheral nerves.^12^

**Examination**: low tone and inferior subluxation are evident on examination.

**Investigations**: key x-ray findings include inferior subluxation of the humeral head.

Ultrasound may demonstrate rotator cuff tears or tendinosis. It is worth noting that rotator cuff tears are commonly found in the general population, with asymptomatic tears twice as common as symptomatic tears.^31^ Thus, the finding of a rotator cuff tear on imaging does not necessarily mean that this is the cause of the patient’s pain.

**Spasticity**

**History**: spasticity may be associated with pain and/or stiffness. Spasticity may also increase in the presence of pain.

**Examination**: the presence of velocity-dependant increase in tone of the proximal shoulder muscles, commonly but not exclusively seen in the internal rotator and adductor muscles (pectoralis major, subscapularis, teres major and latissimus dorsi). Spasticity severity can be recorded using the Ashworth scale^32^ or the Modified Ashworth Scale.^33^

Associated clinical signs may include internal rotation and adduction of the humerus, and anterior subluxation of the humeral head.

**Investigations**: x-ray may reveal anterior dislocation of the humeral head.

**Sub-acromial pain syndrome (SAPS)**

**History**: pain is typically worse during or just after lifting the affected arm.^16^

**Examination**: malalignment of the shoulder joint may be evident at rest and/or during movement. Poor co-ordination of the scapula in relation to the humerus (scapulohumeral rhythm) may be observed.

Pain is worse during or just after lifting the arm.^16^ Shoulder special tests may be used with caution.

**Investigations**: x-ray can determine presence of co-existing osteoarthritis or osseous abnormalities.^16^ Ultrasound may reveal abnormalities such as rotator cuff tendinopathy and/or tears, bursitis or biceps tendinopathy.

**Frozen shoulder**

**History:** the primary symptom may be pain (pain predominant frozen shoulder) or stiffness (stiffness predominant frozen shoulder).^22^ but often both are present.

**Examination**: gradual loss of both active and passive range of motion (PROM), particularly of external rotation, is evident. At the end of the available PROM, there may be a firm feeling. Pain is brought on by the stretch of the contracted joint capsule.

**Investigations**: an x-ray should be conducted to rule out skeletal pathology that may account for stiffness such as osteoarthritis and heterotopic ossification.^30^ In the presence of a normal x-ray, if external rotation is reduced, frozen shoulder should be suspected.^21^

Key ultrasound findings of frozen shoulder include diminished or absent axillary recess, a thickened capsule and/or irregular capsular margins.^34,35^

## Box 3 Protective handling and positioning of the affected arm

**General recommendations**

▪ In patients with proximal upper limb weakness, the arm must be supported throughout the day. Positioning must promote a neutral alignment.
▪ The patient should be encouraged to change the position of the arm regularly (Figures 2 and 3) to prevent stiffness, as this can precipitate the development of further pain.
▪ Pillows, cushions, a table, or an armrest on a wheelchair which has been provided by a therapist (Figure 4) may be used.
▪ If the patient is changing position, the carer should support the upper arm or forearm (Figure 5).
▪ The arm should never be pulled.

**The use of an upper limb orthosis**

▪ In an ambulant patient, a trained therapist should assess whether an upper limb orthosis for transfers and mobilising is required.^36,37^
▪ The decision to use an orthosis requires careful consideration as it may cause harm if used incorrectly. For example, if a patient has preserved motor activity in the affected limb, the orthosis may limit arm movement and lead to secondary disuse weakness, causing loss of function and potentially further pain.
▪ The suitability of the orthosis should be reviewed regularly.

## Box 4 Management of the four common clinical presentations

**Table:**

**Hypotonia and subluxation**	**Spasticity**
***First line treatment:*** ⍰ Physiotherapist-led interventions: these may include; strengthening exercises for those with preserved upper limb proximal muscle activity, NMES ^38^ and kinesiology taping.^39^This is the application of kinesiology tape to optimise the alignment of the shoulder. ***Second line treatment:*** ⍰ Consider targeted steroid injection if damage to soft tissues with inflammation is present that may account for pain. ***Third line treatment:*** ⍰ Refer to the orthopaedic team for consideration of surgical intervention. However, the benefit of surgery in the management of the flaccid inferiorly subluxed GHJ is questionable. Techniques proposed in the literature include suspension of the humeral head with the coracoacromial ligament or long head of biceps tendon, and arthrodesis.^40^	***First line treatment:*** ⍰ Identify and address all remediable aggravating factors ^41^ such as pain, skin breakdown, or poorly-fitting orthotics. ⍰ Commence a therapist-led positioning programme aiming to reduce the risk of developing contracture, which may precipitate development of further pain. For example, if spasticity is present in the shoulder internal rotator muscles, positioning of the arm in external rotation should be conducted regularly. ***Second line treatment:*** ⍰ Refer to the local spasticity team for a multi-disciplinary assessment. ⍰ Botulinum toxin to the affected muscles will be considered. The onset of treatment effect following botulinum toxin varies but is commonly around 10 days, while peak toxin effect is 3-6 weeks. Its effect usually wears off after 3 months, although this may be longer lasting. ⍰ Appropriate consent must be sought if botulinum toxin is offered, including the risk of causing unintended weakness of the muscles injected and destabilising the shoulder joint. ⍰ If botulinum toxin injection is administered, the assessment of spasticity and physical management plan should be reviewed by the therapy team within 7-14 days.^41^ ⍰ If spasticity is more generalised (e.g., in the upper and lower limb), oral medications such as baclofen or gabapentin may be considered. ⍰ If neuropathic pain is a dominant symptom alongside spasticity, gabapentin or pregabalin may be helpful.^42^ ***Third line treatment***: ⍰ Refer to the orthopaedic team for consideration of surgical intervention. Adduction and internal rotation deformities can be addressed by open or arthroscopic release of the GHJ. This may be considered if the restricted movement is causing pressure ulcers or interfering with axillary hygiene.
**Sub-Acromial Pain Syndrome (SAPS)**	**Frozen shoulder**
***First line treatment:*** ⍰ Physiotherapist-led interventions: commence an individualised, supervised and progressed exercise programme. ^43,44^ Consider adjuncts such as kinesiology taping. ***Second line treatment:*** ⍰ Consider targeted steroid injection if inflammation present which may account for pain, e.g., in the subacromial bursa. ***Third line treatment*:** ⍰ Referral to the orthopaedic team for consideration of surgical intervention, such as arthroscopic subacromial decompression with/without acromioplasty. ⍰ If a chronic full thickness rotator cuff tear has been identified, open or arthroscopic repair may be considered if symptoms are refractory to conservative measures. However, chronic full thickness tears associated with muscle retraction and fatty infiltration may not be amenable to surgical repair. A radiological finding of rotator cuff tear or bursal thickening is not an indication for surgery in itself.	***First line treatment:*** ⍰ Physiotherapist-led interventions, which aim to reduce pain and increase range of movement. These may include; stretching exercises up to a pain-tolerable range of motion,^45^ strengthening exercises for those with preserved proximal upper limb muscle activity,^46^ joint mobilisations^46^ and neuromuscular electrical stimulation (NMES) if no contraindications to its use are present.^38^ NMES is the application of an electrical current usually via transcutaneous pads to elicit a muscle contraction. *Note: It is important to grade interventions appropriately, considering the severity of pain.* **Second line treatment:** ⍰ Consider intra-articular corticosteroid injection. The provision of physiotherapy promptly following corticosteroid injection may confer added benefit. ^47^ ⍰ In stiffness predominant presentation, hydrodilatation may be of benefit.^48^ Hydrodilatation is the injection of fluid (usually saline and local anaesthetic) into the GHJ (the technique and injection volume vary widely). This may be combined with intra-articular corticosteroid injection. ***Third line treatment:*** ⍰ Refer to the orthopaedic team for consideration of surgical intervention. This may involve manipulation under anaesthetic or arthroscopic capsular release.^49^

## Box 5 Pain management considerations

**Physical**

Consider heat packs in frozen shoulder.^52^ Consider acupuncture, particularly if Shoulder-Hand Syndrome (SHS) is present.^53^

**Medical**

The medication prescribed will depend on the type(s) of pain the patient has: nociceptive, neuropathic, or a combination.

**Nociceptive pain:**

A step-wise approach to managing nociceptive pain should be used, escalating treatment if it proves to be insufficient. Medication can be provided on a regular basis and/or as required, depending on the clinical requirements of the patient.

**Mild pain:** may be treated with non-opioid medication (e.g.: paracetamol) and/or an anti-inflammatory agent (e.g.: ibuprofen). Anti-inflammatory agents in this patient group are generally only suitable for short-term use, and in many cases may be contra-indicated.

**Moderate pain:** consider the addition of a weak opioid medication (e.g.: codeine). Constipation as a side effect is common, so laxatives may be considered in conjunction.

**Severe pain:** stronger opiates (e.g.: oxycodone) may be considered, however should be used with caution.^54^ Only prescribe strong opioids in instances of severe, acute (<12 weeks) pain. In patients with neurological conditions, they may be particularly at risk of cognitive and/or psychiatric side effects.

**Neuropathic pain:**

For the treatment of neuropathic pain, we recommend following the guidelines from the National Institute of Clinical Excellence:^42^

⍰ Amitriptyline, duloxetine, gabapentin or pregabalin may be offered as initial treatment.
⍰ If the initial treatment is not effective or is not tolerated, offer one of the remaining 3 drugs.
⍰ Consider switching again if the second and third drugs tried are also not effective or not tolerated.
⍰ It is important to be aware that the combination of gabapentin and opioids may be associated with increased adverse events, including a reduced level of consciousness and respiratory depression. If combining duloxetine and amitriptyline one must also be aware of the risk of developing the serotonin syndrome at higher doses.

**Any:**

**Suprascapular nerve block (SSNB):**

SSNB under ultrasound guidance may also be considered at any point in the patient’s care provided there are goals for treatment. SSNB may consist of local anaesthetic, steroid, or a combination of both. Evidence suggests it is a safe procedure and can both reduce pain and increase range of movement of the shoulder.^55,56,57^ It can be used to treat pain in the shoulder of any type and there is early evidence to suggest SSNB may be helpful in treating acute pain related to SHS.^58^ It may also be used to assist in the provision of other more painful procedures such as hydrodilatation.

As well as supplying around 70% of the sensory fibres to the shoulder, the suprascapular nerve also contributes to motor function of the shoulder. A block should therefore be considered with guidance from a rehabilitation specialist. If successful, longer-lasting procedures such as pulsed radiofrequency or phenol could be considered in specific cases.

**Psychological**

A referral to psychology services should be considered in all cases.

## Key points

⍰ Shoulder pain in patients with neurological conditions requires multi-disciplinary and multifaceted assessment.
⍰ It tends to present as one or more of four clinical presentations: hypotonia with subluxation, spasticity, sub-acromial pain syndrome (SAPS) and frozen shoulder. Nociceptive and/or neuropathic pain may be present.
⍰ Each presentation has unique clinical features and requires specific physical and medical management strategies.
⍰ The mainstay of treatment of all presentations is conservative via the use of positioning and handling guidelines and physical therapies.
⍰ Steroid injections may be considered primarily for the management of acute exacerbations of pain not responding to first line treatments and simple analgesia, or in the context of meeting particular functional goals in the rehabilitation setting and should not be considered routinely. Repeated injection is not generally recommended.
⍰ Supra-scapular nerve block may be considered at any point following the diagnosis of shoulder pain providing there are goals for treatment.
